# Cardiopulmonary Exercise Testing in Chronic Obstructive Pulmonary Disease: A Narrative Review

**DOI:** 10.3390/healthcare14213721

**Published:** 2026-11-02

**Authors:** Debapriya Datta, Richard ZuWallack

**Affiliations:** 1 Division of Pulmonary and Critical Care Medicine, University of Connecticut Health Center, 263 Farmington Avenue, Farmington, CT 06030, USA; 2 Division of Pulmonary and Critical Care Medicine, St Francis Hospital & Medical Center, Hartford, CT 06105, USA

**Keywords:** CPET, COPD, dyspnea, dynamic hyperinflation, exercise, exercise limitation, exercise testing, functional capacity

## Abstract

Chronic obstructive pulmonary disease (COPD) is characterized by airflow limitation from the narrowing of small airways and damage to lung parenchyma, resulting in exercise intolerance. Cardiopulmonary exercise testing (CPET) provides a comprehensive assessment of exercise capacity and of the integrated responses of the respiratory, cardiovascular, muscular and metabolic systems. It helps clinicians understand the mechanisms of dyspnea, informs treatment decisions and can evaluate the effectiveness of pulmonary rehabilitation and other treatments. CPET can also detect coexisting conditions, such as heart failure, myopathy or deconditioning, that worsen exercise intolerance in COPD despite treatment. Recognizing these comorbidities allows them to be investigated and treated in their own right. Because CPET is resource-intensive, it is best used selectively, as a phenotyping and problem-solving tool, and it is important that pulmonologists know when to request it and how to interpret it. In this review, the mechanisms of exercise limitation in COPD and the physiological measurements obtained during CPET are described, and the CPET patterns that distinguish ventilatory limitation from cardiac, pulmonary vascular and other causes of exercise intolerance are compared. Testing protocols, the interpretation of results in relation to bronchodilator therapy, and safety and contraindications are summarized. Developments since previous reviews and guidelines on COPD and lung cancer fitness, as well as recent evidence on ventilatory efficiency and prognosis are highlighted, with emphasis on integrative interpretation. Clinical applications are illustrated with an algorithm and a case example, and limitations, cost, barriers to implementation and future research are discussed.

## 1. Introduction

Chronic obstructive pulmonary disease (COPD) is characterized by airflow limitation from the narrowing of small airways, causing increased airway resistance, and damage to the lung parenchyma, causing reduced elastic recoil. These abnormalities result in exercise limitation, shortness of breath and functional impairment. COPD, which includes emphysema and chronic bronchitis, is a progressive, life-threatening lung disease [1]. The overall prevalence of COPD in people aged ≥40 years was 12.64% (95% CI 10.75–14.65%) [2]. COPD is the fourth leading cause of death worldwide, causing 3.5 million deaths in 2021, approximately 5% of all global deaths [2].

COPD is the eighth leading cause of poor health worldwide, measured by disability-adjusted life years [2]. Although COPD is not curable, several interventions can reduce symptoms and improve outcomes in appropriately selected patients. These include smoking cessation, inhaled pharmacological therapy, long-term oxygen therapy in patients with severe resting hypoxemia, vaccination, reduction of exposure to pollutants, and pulmonary rehabilitation (PR) [1,3,4,5].

In a modeling study of future COPD burden, the number of COPD cases globally among people aged ≥25 years was estimated to increase by 23% from 2020 to 2050, approaching 600 million patients with COPD globally by 2050 [6]. This growing burden makes it important to characterize the causes of symptoms in individual patients accurately and to use diagnostic resources efficiently. Cardiopulmonary exercise testing (CPET) can contribute to this.

Several comprehensive reviews of CPET in COPD have been published, most recently by Behnia and Sietsema in 2023 [7], and others have addressed exertional dyspnea and the physiological basis of CPET interpretation [8,9]. The present review does not aim to redescribe established exercise physiology in detail. Instead, it updates the clinical use of CPET in COPD in three ways. First, it integrates guidance and evidence published since those reviews: the Global Initiative for Chronic Obstructive Lung Disease (GOLD) 2026 report, which positions exercise testing as an additional investigation when symptoms and airflow obstruction are discordant [3]; the 2025 European Respiratory Society (ERS) and European Society of Thoracic Surgeons (ESTS) guideline on fitness for curative-intent treatment of lung cancer [10]; recent work on ventilatory efficiency (the relationship between minute ventilation and carbon dioxide output, VE/VCO_2_, expressed as slope, nadir and intercept) [11]; and a 2026 systematic review of the prognostic value of CPET variables [12]. Second, it emphasizes integrative interpretation over fixed cut-offs, and it distinguishes physiological interpretation from evidence that CPET-guided management improves patient outcomes. Third, it offers practical guidance for the general pulmonologist on when to request CPET rather than a field test, on safety and contraindications, on testing in relation to bronchodilator therapy, and on the limitations and cost of the test. These developments are summarized in Table 1.

Despite this body of work, important gaps remain. CPET practice and interpretation vary considerably between laboratories [15,16]. Guidance on when CPET adds value beyond simpler field tests is dispersed across respiratory, cardiology and thoracic surgery documents, and the extent to which CPET findings should change management in COPD has rarely been appraised critically. The aim of this narrative review is to summarize the physiological basis of exercise limitation in COPD and the CPET variables used to characterize it; to describe how CPET patterns distinguish ventilatory limitation from other causes of exercise intolerance; and to critically appraise the evidence for the clinical applications of CPET in COPD, with a focus on when, and how, the test can inform management.

## 2. Methods

This is a narrative review. We searched PubMed/MEDLINE from January 2000 to April 2026 for English-language articles, using combinations of the terms “cardiopulmonary exercise test*”, “exercise test*”, “COPD”, “chronic obstructive pulmonary disease”, “dynamic hyperinflation”, “ventilatory efficiency”, “VE/VCO2”, “oxygen uptake efficiency slope”, “dyspnea”, “pulmonary rehabilitation”, “lung resection” and “preoperative”. Older publications were included where they remain the primary source for physiological concepts or reference values. We gave priority to, in order: official guidelines and statements (GOLD, American Thoracic Society [ATS], ERS, ESTS, American College of Chest Physicians [ACCP], International Society for Heart and Lung Transplantation [ISHLT]); systematic reviews and meta-analyses; large cohort studies and randomized trials; and narrative reviews from established research groups. Reference lists of key articles were hand-searched. No formal quality assessment was performed. The core search string, adapted to each database, was as follows: (“cardiopulmonary exercise test*” OR “CPET” OR “CPX” OR “exercise test*”) AND (“COPD” OR “chronic obstructive pulmonary disease” OR “emphysema”) AND (“dyspnea” OR “dyspnoea” OR “dynamic hyperinflation” OR “oxygen uptake efficiency slope” OR “heart failure” OR “pulmonary rehabilitation” OR “preoperative”). We included guidelines, official statements, systematic reviews, randomized and non-randomized clinical studies, and narrative reviews. We excluded case reports, conference abstracts, non-English publications and pediatric studies. Evidence was selected according to its relevance to the four areas addressed by the review: physiology and measurement, interpretation patterns, clinical applications, and limitations and implementation.

## 3. Assessing COPD

Pulmonary function tests (PFTs) are routinely used to diagnose COPD and evaluate its severity [1]. According to the GOLD 2026 report, COPD is confirmed in the appropriate clinical context by a post-bronchodilator ratio of forced expiratory volume in one second (FEV_1_) to forced vital capacity (FVC) of less than 0.70, and severity is graded from mild to very severe based on post-bronchodilator FEV_1_ as a percentage of predicted [1,3]. Other PFT abnormalities in COPD include a reduced diffusing capacity of the lung for carbon monoxide (DLCO), reflecting loss of alveolar surface area for gas exchange [17]; an increased total lung capacity (TLC), indicating hyperinflation; and an increased residual volume (RV), indicating air trapping.

**Figure 1:**
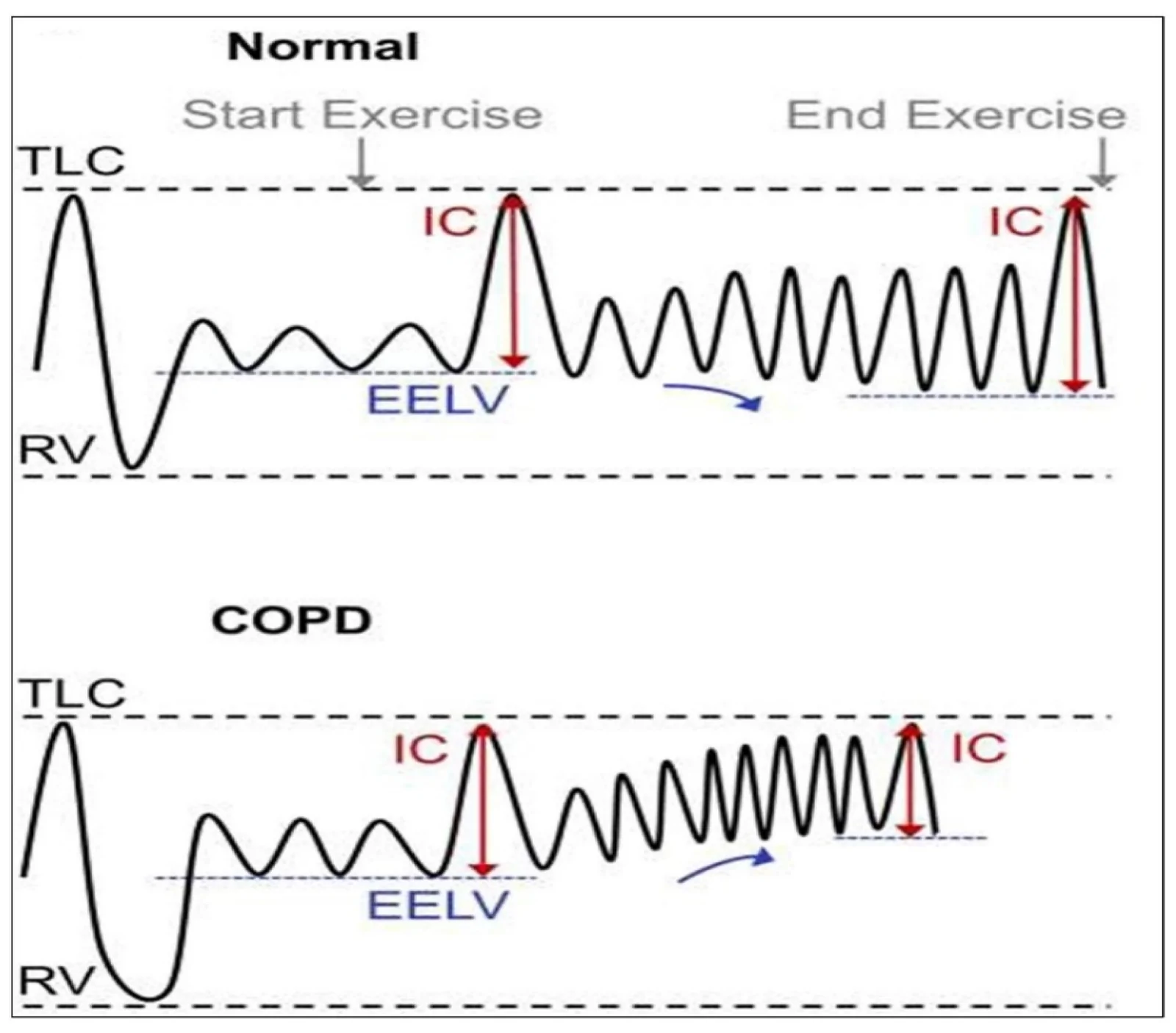
Spirogram at rest and during exercise in a healthy subject and in a patient with COPD. In the healthy subject EELV decreases during exercise, allowing VT to expand; in COPD, EELV increases (dynamic hyperinflation), reducing IC and constraining VT expansion. Abbreviations: EELV, end-expiratory lung volume; IC, inspiratory capacity; RV, residual volume; TLC, total lung capacity; VT, tidal volume. Source: [18]. Reprinted with permission of the American Thoracic Society. Copyright © 2006 American Thoracic Society.

End-expiratory lung volume (EELV) is the volume of air remaining in the lungs at the end of a tidal breath; in a spontaneously breathing person at rest it corresponds to the functional residual capacity (FRC) [18]. In COPD, increased lung compliance and reduced elastic recoil, together with expiratory airflow limitation, raise TLC and EELV, and incomplete exhalation elevates RV (Figure 1) [18,19]. How these resting volumes change during exercise is described in the next section.

PFTs are performed at rest and do not directly assess the exertional dyspnea that is common in COPD, and exertional symptoms and exercise capacity often correlate poorly with resting measurements such as FEV_1_ [8,9,20]. Exertional dyspnea in COPD is closely related to dynamic hyperinflation (described in the next section) and the resulting mechanical constraint on tidal volume expansion [18], and to ventilatory inefficiency, particularly in milder disease [8,11].

GOLD 2026 accordingly suggests that additional assessment, including lung volumes, diffusing capacity, exercise testing or imaging, may be considered when the level of airflow obstruction and perceived symptoms are markedly discordant [3]. Measurements made during exercise can therefore provide information about functional capacity and the mechanisms of exertional symptoms that cannot be obtained from testing at rest [9,20,21]. In addition, dysfunction in COPD may not be confined to the lung, and testing the body’s overall physiological response to exercise provides useful data about other organs, such as the heart or skeletal muscle, that may contribute to exercise limitation.

## 4. Physiology of Exercise Impairment and Hypoxemia During Exercise in COPD

Expiratory flow limitation, the underlying pathophysiological mechanism of COPD, arises from the combined effects of reduced elastic recoil and increased airway resistance [22,23]. In healthy subjects, respiratory rate (RR) and tidal volume (VT) increase during exercise to meet metabolic demand, while EELV decreases [24]. In COPD, expiratory flow is limited even at rest, so the time available for expiration may be insufficient for the lungs to return to their relaxation volume before the next inspiration begins. The result is hyperinflation and air trapping [22,25]. During exercise, the rise in RR shortens the time available for expiration, and EELV rises further [18,22,25]. This exercise-induced increase in EELV above its resting value is termed dynamic hyperinflation (DH) [25]. It is the principal mechanical abnormality of exercise in COPD, and the remainder of this review refers back to this description.

DH can occur even in mild COPD and in patients without hyperinflation or air trapping on resting PFTs [18,25,26]. With expiratory flow limitation, the lungs do not empty to their relaxation volume before the next inspiration begins, so alveolar pressure remains positive at end expiration. This is intrinsic positive end-expiratory pressure (PEEPi). The inspiratory muscles must generate enough pressure to overcome PEEPi before inspiratory flow can begin, which imposes an inspiratory threshold load. As EELV rises further with DH, tidal breathing is displaced toward TLC, onto the less compliant upper portion of the pressure–volume curve. The elastic work of breathing therefore increases, the inspiratory muscles operate at a shorter, mechanically disadvantaged length, VT expansion becomes constrained, and inspiratory capacity (IC) falls. Together these changes increase the work of breathing and dyspnea. The associated rise in intrathoracic pressure can also reduce venous return (preload) and, in severe cases, cause hypotension [26,27]. The significance of DH is demonstrated by studies in which exercise performance improves when hyperinflation is reduced, for example with bronchodilator therapy [14,28]. Bronchodilators act by improving expiratory airflow and lung emptying, which lowers operating lung volumes; they do not act by changing expiratory time.

Hypoxemia in COPD results from ventilation–perfusion (V/Q) mismatch, diffusion limitation and impaired ventilatory function [29]. During exercise, V/Q mismatch may worsen, diffusion impairment increases as red-cell transit time through the pulmonary capillaries shortens, and mechanical ventilatory limitation can cause relative alveolar hypoventilation. Because patients may have normal oxygen saturation at rest but desaturate with exertion, and exertional desaturation cannot be predicted reliably from resting measurements, an exercise assessment with oximetry (usually a 6 min walk test) is commonly used when ambulatory oxygen is being considered [30,31]. Current ATS guidance suggests ambulatory oxygen for adults with COPD who have severe exertional hypoxemia, as a conditional recommendation based on low-quality evidence [5].

## 5. Exercise Testing in COPD

Exercise testing is useful in COPD in numerous ways. Apart from evaluating the need for oxygen during activity, it quantifies functional capacity and can detect other factors contributing to exercise intolerance, such as coexisting cardiac or neuromuscular dysfunction or deconditioning [32]. Exercise tests used in COPD fall into two broad groups: field walking tests and laboratory tests. This review considers three: the six-minute walk test (6MWT), shuttle walk test (SWT) and CPET. These are the tests addressed by international technical standards for chronic respiratory disease, with established protocols and measurement properties [13,14,31].

The 6MWT measures the distance a person can walk in six minutes at a self-selected pace (six-minute walk distance, 6MWD). It is a low-technology test that is easily performed and helps determine functional capacity [33]; the 6MWD can be used to assess response to treatment and disease progression in COPD [34]. The SWT is an externally paced test performed on a 10 m course [31]. It is an incremental test in which the patient walks between two cones 10 m apart, following audio signals whose pace increases every minute, for as long as possible [35]. The shuttle walk distance (SWD) is also an indicator of functional capacity and can be used to assess response to treatment and disease progression. Both field tests are inexpensive and reliable, but a learning effect means that two tests are needed at baseline [14].

**Figure 2:**
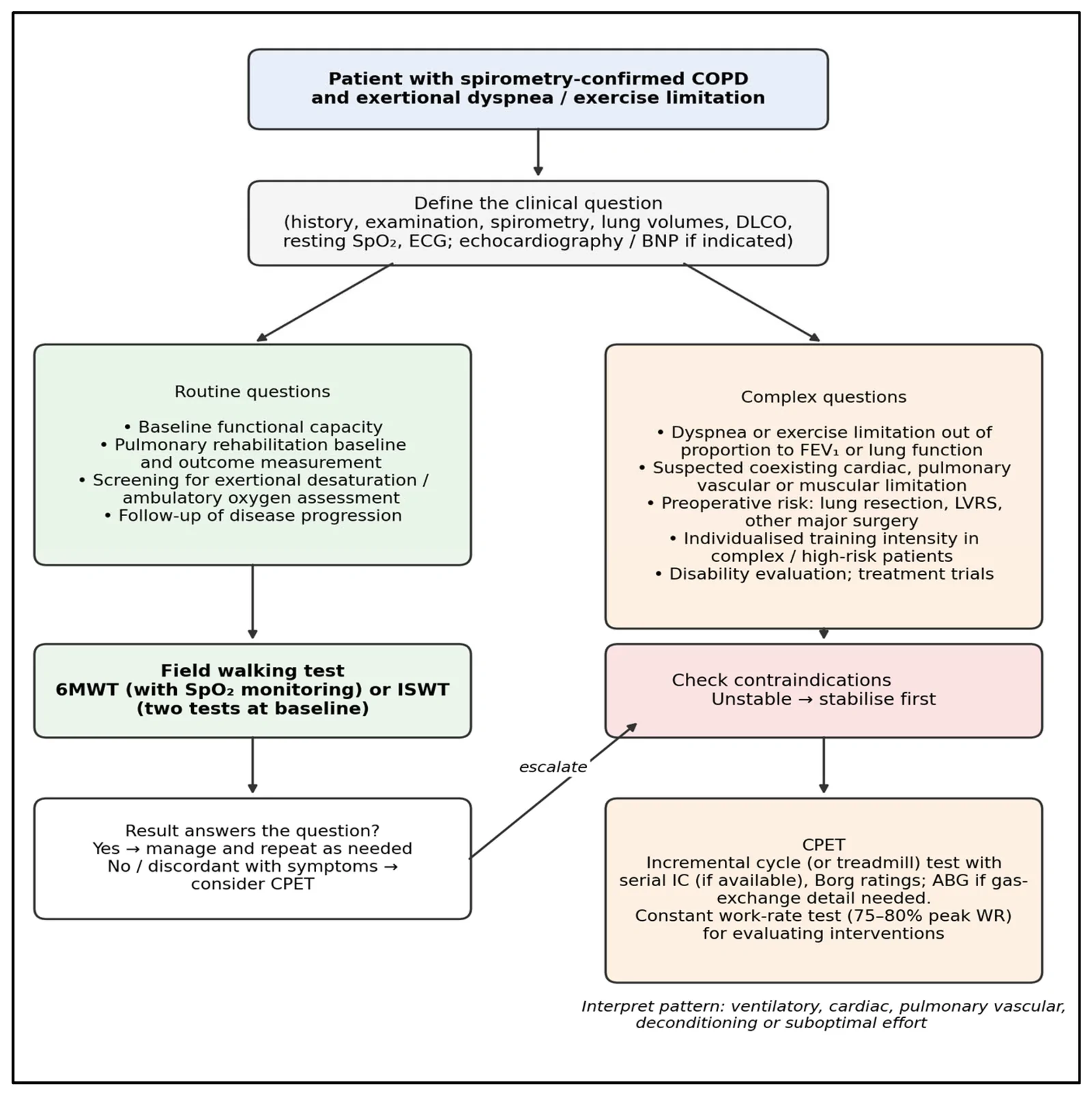
Suggested clinical algorithm for choosing between field walking tests and CPET in COPD. Abbreviations: 6MWT, six-minute walk test; ABG, arterial blood gas; BNP, B-type natriuretic peptide; DLCO, diffusing capacity for carbon monoxide; ECG, electrocardiogram; FEV_1_, forced expiratory volume in one second; IC, inspiratory capacity; ISWT, incremental shuttle walk test; LVRS, lung volume reduction surgery; SpO_2_, pulse oximetric saturation; WR, work rate. Original figure created by the authors. The algorithm reflects the authors’ synthesis of GOLD 2026 [3], ERS statements [13,14] and the ERS/ESTS guideline [10]; it has not been prospectively validated.

CPET measures the physiological responses to exercise on a treadmill or cycle ergometer, including heart rate (HR), blood pressure (BP), oxygen saturation, RR, VT, minute ventilation (VE), oxygen uptake (VO_2_) and carbon dioxide output (VCO_2_) [36]. Peak VO_2_ is an accurate indicator of functional capacity and can be used to guide therapeutic interventions, assess their effectiveness and monitor disease progression. Field tests measure distance walked or time, and, with oximetry, heart rate and oxygen saturation. CPET additionally measures ventilation and gas exchange breath by breath. This allows the anaerobic threshold (AT), ventilatory efficiency, breathing reserve (BR) and oxygen pulse (O_2_ pulse) to be determined, and therefore allows the system or systems limiting exercise to be identified [20,37]. Field tests, in turn, more closely reflect everyday walking and require less equipment [31]. Field tests are sufficient for many routine questions, whereas CPET is reserved for questions that require mechanistic information; a suggested approach is shown in the clinical algorithm in Figure 2. CPET is therefore not a routine investigation for every patient with COPD [3].

## 6. Cardiopulmonary Exercise Test (CPET)

CPET is a non-invasive exercise test that evaluates the integrated response of the heart, lungs and muscles to exercise. Patients exercise on a treadmill or cycle ergometer while the workload is progressively increased until symptoms (such as dyspnea, leg fatigue, chest pain or dizziness) or signs (such as arrhythmia, ST-segment changes or a fall in BP) limit continued exercise. Inspired and expired gases are collected via a mouthpiece or face mask and analyzed breath by breath, and the data are time-averaged to generate combined cardiac, ventilatory and gas exchange variables [13,20,24,37]. CPET thus provides a comprehensive evaluation of the respiratory, cardiovascular, muscular and metabolic responses to exercise [16,20,24,38].

### 6.1. Testing Protocols

Cycle ergometry is generally preferred in COPD because work rate (WR) is measured directly, the patient is more stable for blood sampling and IC maneuvers, and there is less risk of falls; treadmill testing involves a more familiar activity but typically yields a higher peak VO_2_ [13,37]. The European Respiratory Society (ERS) standard for chronic lung disease recommends at least 3 min of resting measurements, 3 min of unloaded pedaling, an incremental phase in which WR increases linearly at least every minute so that the test lasts 8–12 min, and a recovery phase of at least 2–3 min [13]. The WR increment should therefore be individualized, with smaller increments in patients with more severe impairment.

Ratings of dyspnea and leg discomfort on the Borg scale should be obtained during every test. In COPD, the protocol may be supplemented by serial IC maneuvers (for example, every 2 min and at peak exercise) to track EELV and DH, and by analysis of tidal flow–volume loops [9]. These are valuable additional measurements, but they require specific software, staff expertise and patient training, and they are not part of every routine clinical CPET protocol.

Arterial blood gas (ABG) sampling at rest and at peak exercise is needed when an accurate measurement of arterial O_2_ tension (PaO_2_), the alveolar–arterial O_2_ gradient (P(A-a)O_2_) or the dead-space-to-tidal-volume ratio (VD/VT) is required, because pulse oximetry and end-tidal gas tensions are imprecise surrogates in lung disease [37]. For evaluating interventions such as bronchodilators or PR, a constant work rate (endurance) test at 75–80% of the peak WR from a prior incremental test is considerably more responsive than an incremental test or 6MWT [14].

### 6.2. Safety, Contraindications and Monitoring

CPET is a safe procedure when recommended precautions are followed [14]. In a series of 5060 tests in patients with high-risk cardiovascular diseases, adverse events occurred in 0.16% of tests, most commonly ventricular tachycardia, and there were no deaths [39]. Testing should be supervised by trained personnel, with a physician immediately available and resuscitation equipment on hand. Absolute and relative contraindications are listed in Table 2 [37]. In COPD, particular attention should be paid to clinical stability after a recent exacerbation, to resting hypoxemia (the test may be performed with supplemental oxygen, which must be documented and accounted for in interpretation), and to coexisting cardiovascular disease.

Continuous electrocardiographic and pulse oximetry monitoring, with BP measurement at regular intervals, is mandatory. The test should be stopped for chest pain suggestive of ischemia, ischemic electrocardiographic changes, complex ectopy or high-grade atrioventricular block, a fall in systolic BP of more than 20 mmHg from the highest value, marked hypertension (systolic > 250 mmHg or diastolic > 120 mmHg), severe desaturation (SpO_2_ ≤ 80%) with symptoms or signs of severe hypoxemia, sudden pallor, loss of coordination, mental confusion, dizziness or signs of respiratory failure [37].

### 6.3. Interpreting CPET in Relation to Bronchodilator Therapy

For clinical (diagnostic) CPET, patients usually take their regular inhaled medications, so that the test reflects their usual treated state; the drugs and the time of the last dose should be recorded. When serial tests are compared, medication and its timing should be kept constant. Bronchodilators should be withheld only when the clinical question is the effect of bronchodilation itself, in which case the withholding intervals used for spirometry are appropriate [14].

Bronchodilators improve expiratory airflow and lung emptying, and thereby reduce resting and dynamic hyperinflation, increase IC, delay the point at which VT expansion becomes mechanically constrained, and prolong endurance time; the improvement in IC is associated with reduced dyspnea at a given WR [14,28]. Their effect on peak VO_2_ in an incremental test is often small, so changes are best detected by comparing IC and dyspnea at the same exercise time and by endurance time on constant work rate tests [14]. When a patient is tested without bronchodilators, the degree of ventilatory limitation and DH may be greater than in daily life; conversely, ventilatory limitation that persists despite optimal bronchodilation indicates a fixed mechanical constraint and should prompt evaluation of other contributors to dyspnea.

### 6.4. Measurement of Physiological Parameters During CPET [20,24,37,40]

The main CPET variables, their physiological meaning and their relevance in COPD are summarized in Table 3.

1. Functional capacity is estimated by VO_2_ and VCO_2_. Peak VO_2_ (VO_2_peak) is reported when a plateau in VO_2_ (VO_2_max) is not achieved, which is usual in COPD.
2. Ventilatory parameters include maximal voluntary ventilation (MVV), measured at rest before exercise or estimated as FEV_1_ × 35; RR; VT; VE; and breathing reserve (BR). BR is the difference between MVV and peak VE, expressed in L/min (MVV − VEmax) or as a percentage, (1 − VEmax/MVV) × 100. The ventilatory equivalents for CO_2_ (VE/VCO_2_) and O_2_ (VE/VO_2_) are also measured. VE/VCO_2_, expressed as a ratio, as its nadir, or as the slope of VE against VCO_2_, is a measure of ventilatory efficiency, that is, the ventilation required to eliminate a given amount of CO_2_. It is determined mainly by the physiological dead space fraction, VD/VT, and by the set point of the arterial partial pressure of CO_2_ (PaCO_2_). In mild-to-moderate COPD, VE/VCO_2_ is typically increased because of wasted ventilation from V/Q mismatch, and it relates to exertional dyspnea even when FEV_1_ is relatively preserved. In advanced COPD it may appear normal or low (“pseudo-normalized”), because mechanical constraints limit ventilation and CO_2_ is retained [8,11]. The VE/VCO_2_ behaves differently as COPD progresses. The slope tends to fall as mechanical constraints increase. The y-intercept of the VE–VCO_2_ relationship rises. The nadir (lowest value, usually near the anaerobic threshold (AT)) remains high. In advanced COPD, the slope alone can therefore underestimate the severity of gas exchange inefficiency, and the nadir and intercept should be considered alongside it [11].
3. Cardiovascular parameters include HR, BP, the oxygen pulse (O_2_ pulse, VO_2_/HR), heart rate reserve (HRR) and electrocardiographic monitoring for arrhythmia and myocardial ischemia during exercise.
4. Gas exchange parameters include VD/VT, which reflects the degree to which VT is distributed to poorly perfused lung; the end-tidal partial pressures of CO_2_ (PETCO_2_) and O_2_ (PETO_2_); SpO_2_; and, when ABGs are obtained, PaO_2_ and P(A-a)O_2_.
5. Metabolic and integrated parameters.

The AT is the oxygen uptake above which aerobic energy production is supplemented by anaerobic mechanisms, with lactate accumulation and bicarbonate buffering. It reflects an integrated response of O_2_ delivery by the heart, lungs and blood and of O_2_ utilization by skeletal muscle, rather than cardiac function alone. During CPET it is identified non-invasively from gas exchange and ventilatory variables, principally by the V-slope method (the point at which VCO_2_ begins to rise faster than VO_2_), supported by the ventilatory equivalent and end-tidal methods (a rise in VE/VO_2_ and PETO_2_ without a concurrent rise in VE/VCO_2_) [20,24,37]. In COPD, the AT may be indeterminate because ventilatory limitation ends exercise early or because the ventilatory response to buffering is blunted.

The respiratory exchange ratio (RER = VCO_2_/VO_2_) is measured at the mouth and should be distinguished from the respiratory quotient (RQ). The RQ is the ratio of CO_2_ produced to O_2_ consumed at the cellular level, determined by the substrate being metabolized, and the two are equal only in a metabolic steady state. RER is also affected by hyperventilation, by bicarbonate buffering of lactate above the AT, and by CO_2_ retention. No single criterion, whether a peak RER above 1.10 or 1.15, a percentage of predicted maximal heart rate, or a percentage of predicted work rate, establishes adequate effort in every patient. In COPD in particular, exercise is often stopped by ventilatory limitation before RER or heart rate rises substantially. Effort is best judged by integrating several indicators: symptom limitation with high Borg ratings, attainment of a physiological limit in at least one system (for example, VE approaching MVV, heart rate approaching predicted maximum, or a high RER), and the observations of the supervising staff.

### 6.5. Exercise Physiology and Typical Findings on CPET in Healthy Individuals

**Figure 3:**
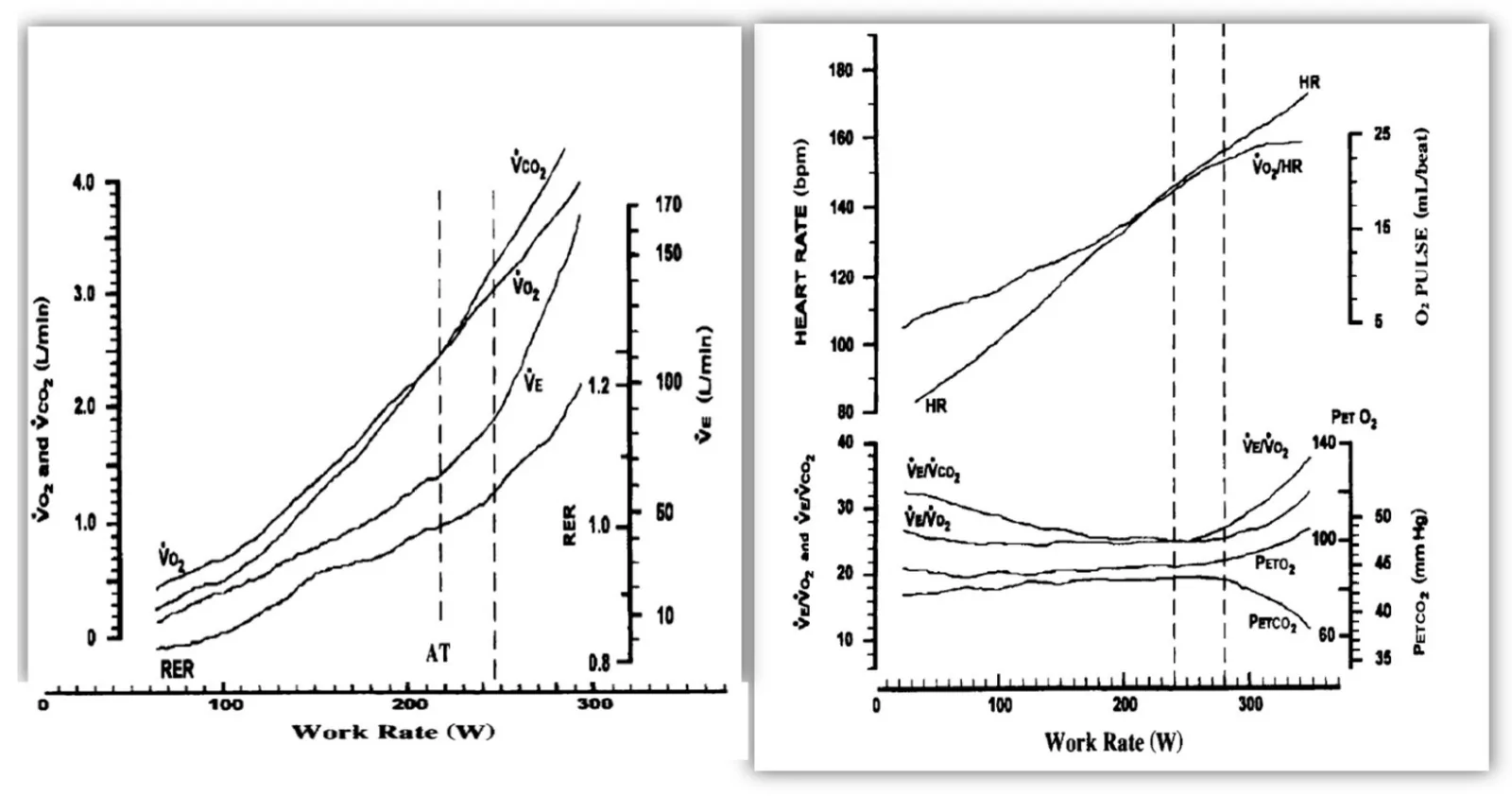
Normal physiologic responses to incremental exercise, showing the changes in heart rate, ventilation, oxygen uptake, carbon dioxide output and gas exchange variables with increasing work rate. Source: Weisman IM, Zeballos RJ. Clinical exercise testing. Clin Chest Med. 2001;22(4):679–701. Reproduced with permission of Elsevier.

With exercise, HR, RR, VT and VE increase as workload increases. VO_2_ and VCO_2_ also increase, rising in parallel until the AT is reached, after which VCO_2_ rises more steeply because of bicarbonate buffering of lactic acid. VD/VT normally decreases, as pulmonary blood flow increases through recruitment and distension of the pulmonary vasculature to accommodate the increased cardiac output [20,24]. The normal physiologic responses to exercise are shown in Figure 3 [36].

In healthy individuals, BR is commonly 15–40% of MVV; individuals with COPD typically have a low BR because of a low MVV together with exaggerated VE requirements.

Commonly cited reference thresholds for physiologic variables measured during CPET are shown in Table 4 [37]. These thresholds are approximations. Normal values depend on age, sex, body size, exercise modality, protocol and the prediction equations used, and lower limits of normal derived from appropriate reference equations are preferable to fixed cut-offs [13,15]. CPET should be interpreted integratively, by examining the pattern of responses across the cardiovascular, ventilatory and gas exchange variables in relation to the patient’s symptoms and the clinical question, rather than by applying isolated thresholds [15].

**Figure 4:**
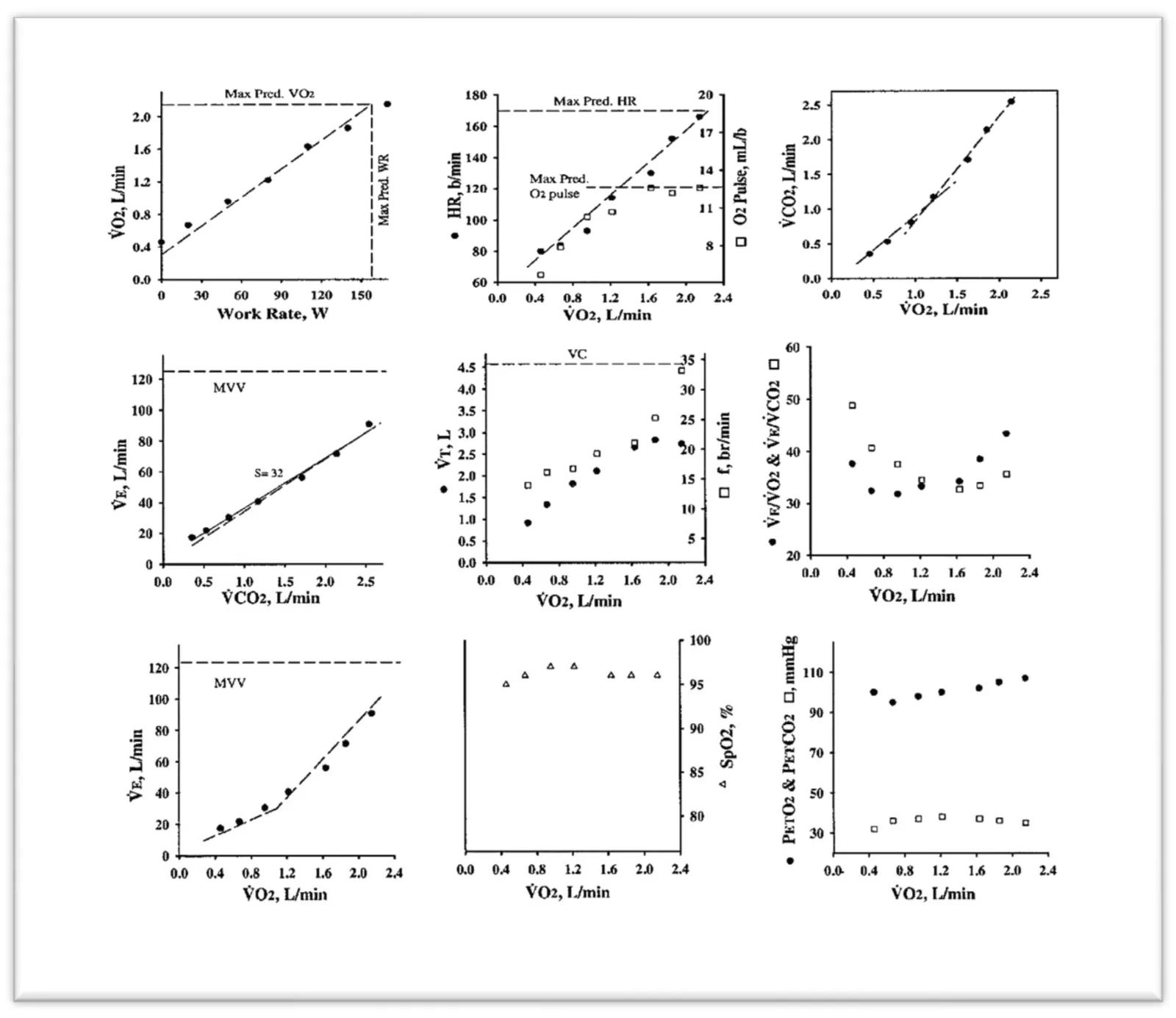
**Nine-panel** graphic display of physiologic variables during incremental exercise in a healthy subject. Panels display ventilatory, cardiovascular and gas exchange variables against time or work rate, and are used as the standard format for CPET interpretation. Source: [37]. Copyright © 2003 American Thoracic Society.

The effect of exercise on these variables in a healthy subject is shown in the nine-panel graphic display in Figure 4 [37].

**Figure 5:**
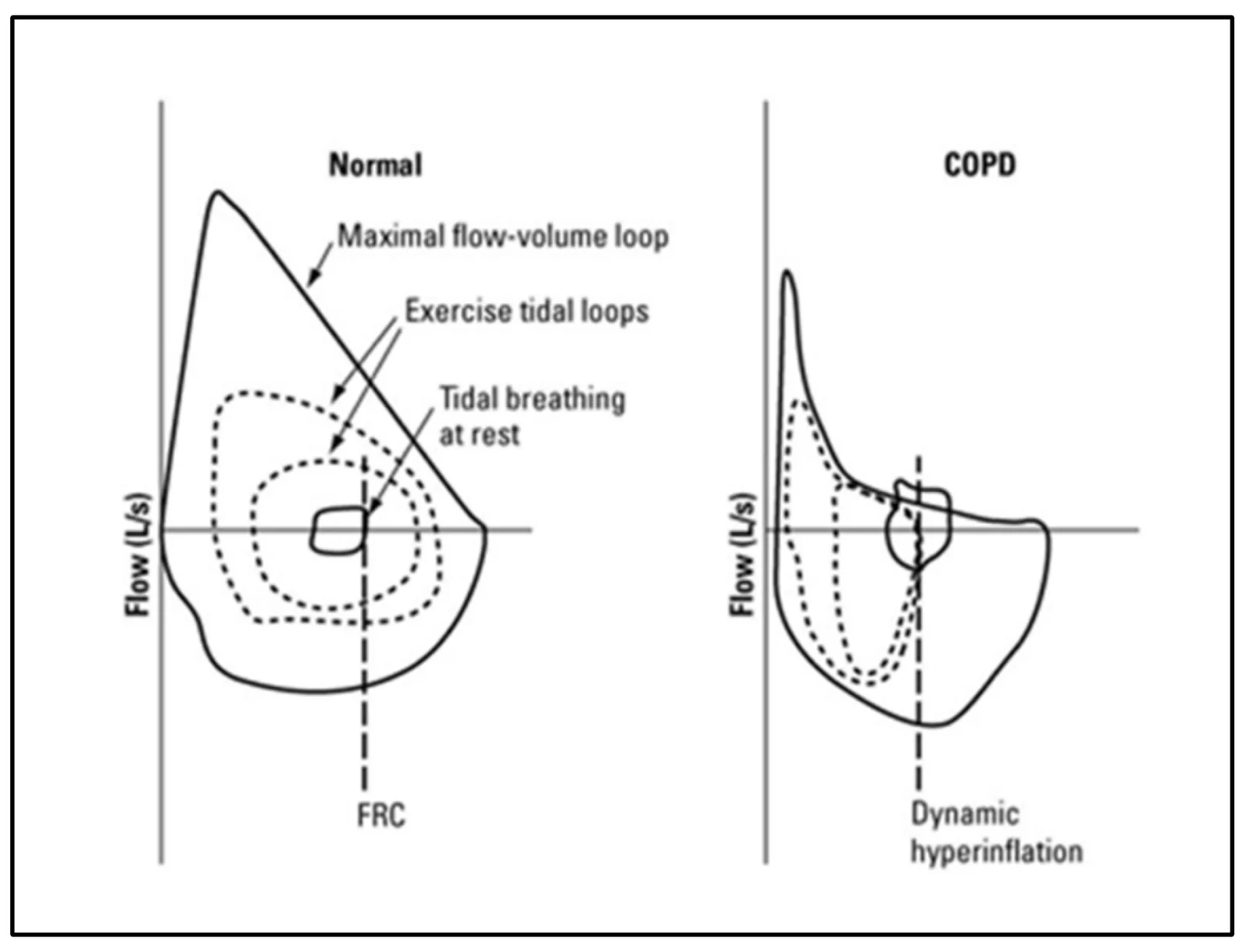
Tidal flow–volume loops at rest and during exercise in relation to the maximal flow–volume loop. In the healthy subject, tidal loops expand in both directions during exercise. In COPD, the shortened expiratory time causes further air trapping and increases EELV, so that the tidal loop shifts to the left (toward TLC), indicating dynamic hyperinflation, and tidal expiratory flow impinges on the maximal expiratory flow envelope (expiratory flow limitation). Abbreviations: EELV, end-expiratory lung volume; TLC, total lung capacity. Source: [19]. Reproduced with permission of the British Columbia Medical Journal.

Respiratory flow is measured continuously during CPET to calculate ventilation and gas exchange. Where the equipment and expertise are available, the tidal flow–volume loops can also be displayed and compared with a maximal flow–volume loop measured before or after exercise (Figure 5) [37]. This shows the extent to which tidal expiratory flow reaches maximal limits [41]. To position tidal loops correctly on the volume axis, IC is measured periodically during exercise; assuming that TLC does not change and that IC maneuvers are performed maximally, a fall in IC reflects an equivalent rise in EELV, that is, DH [40,41].

## 7. Typical Findings on CPET in COPD

**Figure 6:**
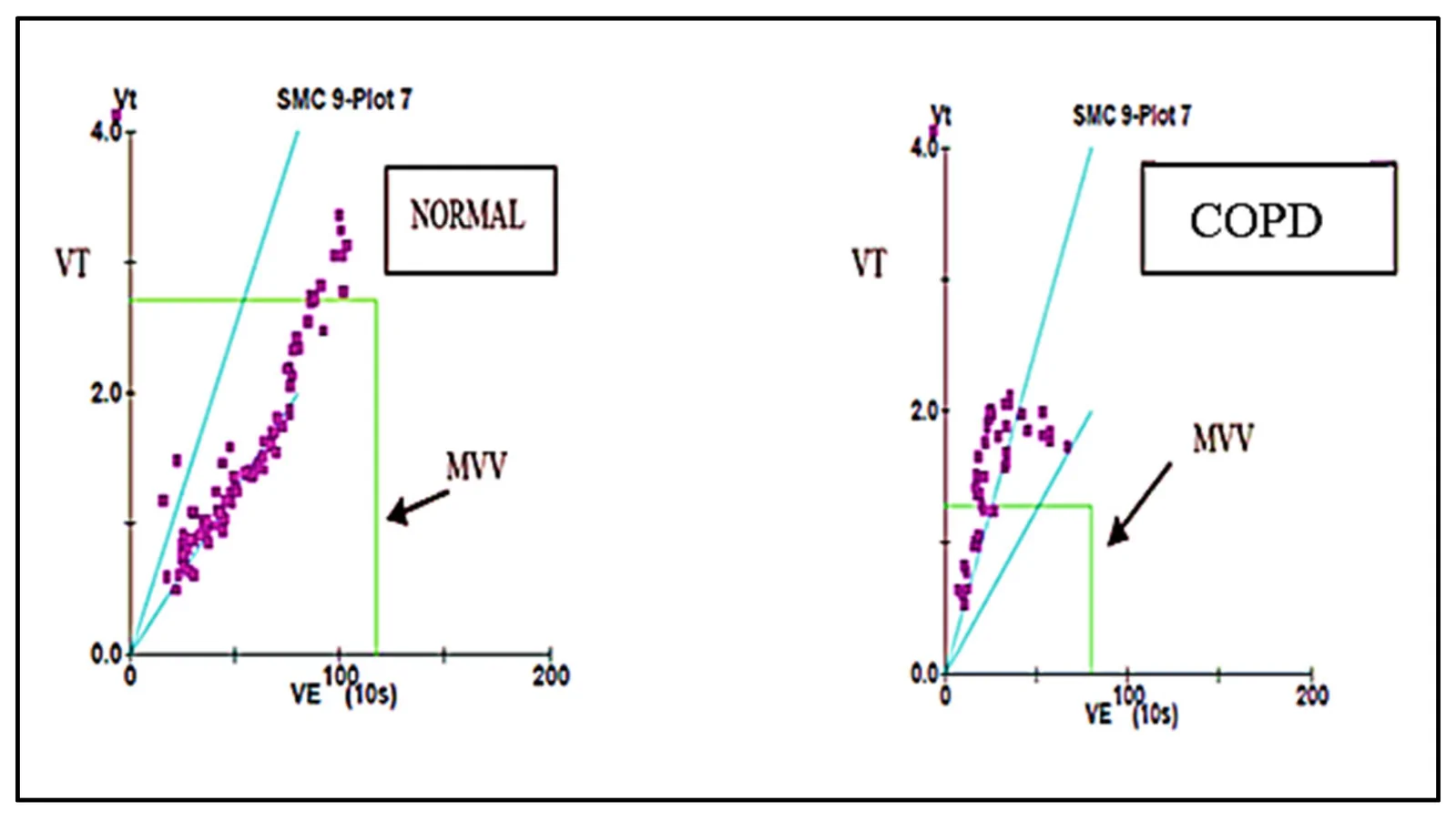
Tidal volume (VT, L) plotted against minute ventilation (VE, L/min) during incremental exercise (panel 7 of the nine-panel display) in a healthy subject (**left**) and a patient with COPD (**right**). The vertical green line marks the maximal voluntary ventilation (MVV). In the healthy subject, peak VE remains well below MVV, leaving a normal breathing reserve. In COPD, MVV is markedly reduced, VT reaches an early plateau, and peak VE approaches or exceeds MVV, indicating ventilatory limitation to exercise. Source: Original tracings from the authors’ exercise laboratory, de-identified.

Patients with COPD typically show features of ventilatory limitation to exercise [9,20,37,42]. VO_2_peak is reduced, indicating reduced functional capacity. MVV is reduced and BR is low or absent, because VE at peak exercise approaches or reaches MVV (Figure 6). RR at maximal exercise may be increased (often > 50 breaths/min, although values up to 60 breaths/min can occur in healthy subjects; Table 4), and hypercapnia may develop.

Cardiovascular responses in COPD may be normal or borderline abnormal [24,37]. Because ventilatory limitation may occur before cardiovascular limitation is reached, the patient may be unable to achieve the predicted maximal HR, so HRR at peak exercise is often increased or normal. For similar reasons, the AT may be normal or may not be reached, and is then indeterminate. The O_2_ pulse is usually normal. Gas exchange abnormalities may be present [11,24]. VD/VT may be increased at rest, with a less-than-normal reduction during exercise, and VE/VCO_2_ and VE/VO_2_ are often increased above the normal range (Table 4), reflecting inefficient ventilation, subject to the caveat about advanced disease described above [11]. PETCO_2_ may be low when dead space ventilation is increased, or may rise toward peak exercise when mechanical constraints cause relative alveolar hypoventilation. Oxygen saturation may fall during exercise.

**Figure 7:**
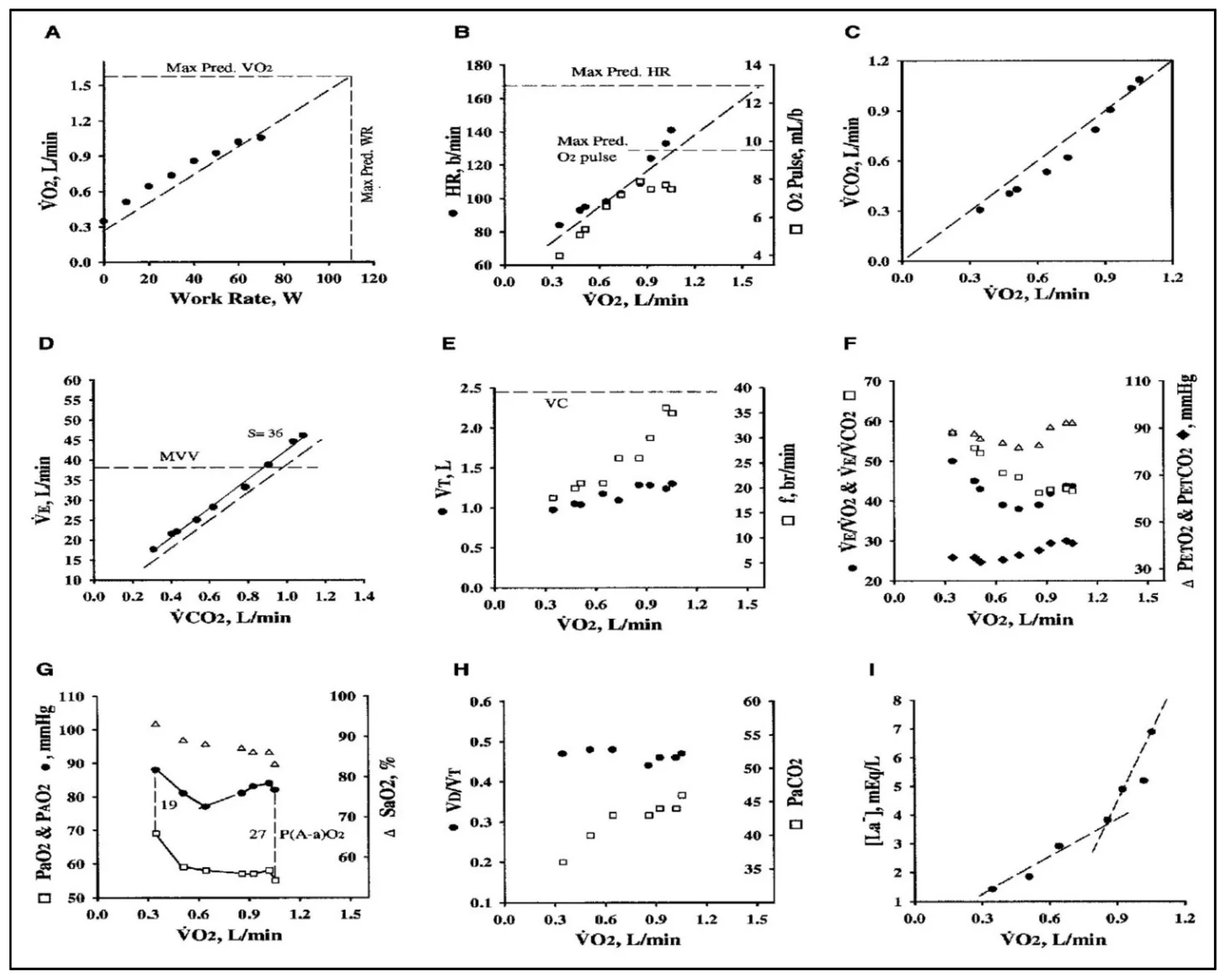
Nine-panel graphic display of physiologic variables during incremental exercise in a patient with COPD, showing features of ventilatory limitation: reduced peak VO_2_, minute ventilation approaching MVV with little breathing reserve, preserved heart rate reserve, and elevated ventilatory equivalents. Source: [37]. Reprinted with permission of the American Thoracic Society. Copyright © 2003 American Thoracic Society.

DH, described above, is identified during CPET when serial IC maneuvers are performed, by a progressive fall in IC, with tidal flow–volume loops shifting toward TLC and impinging on the maximal expiratory flow–volume envelope (Figure 5). The nine-panel display of physiologic variables during CPET in a patient with COPD is shown in Figure 7.

**Figure 8:**
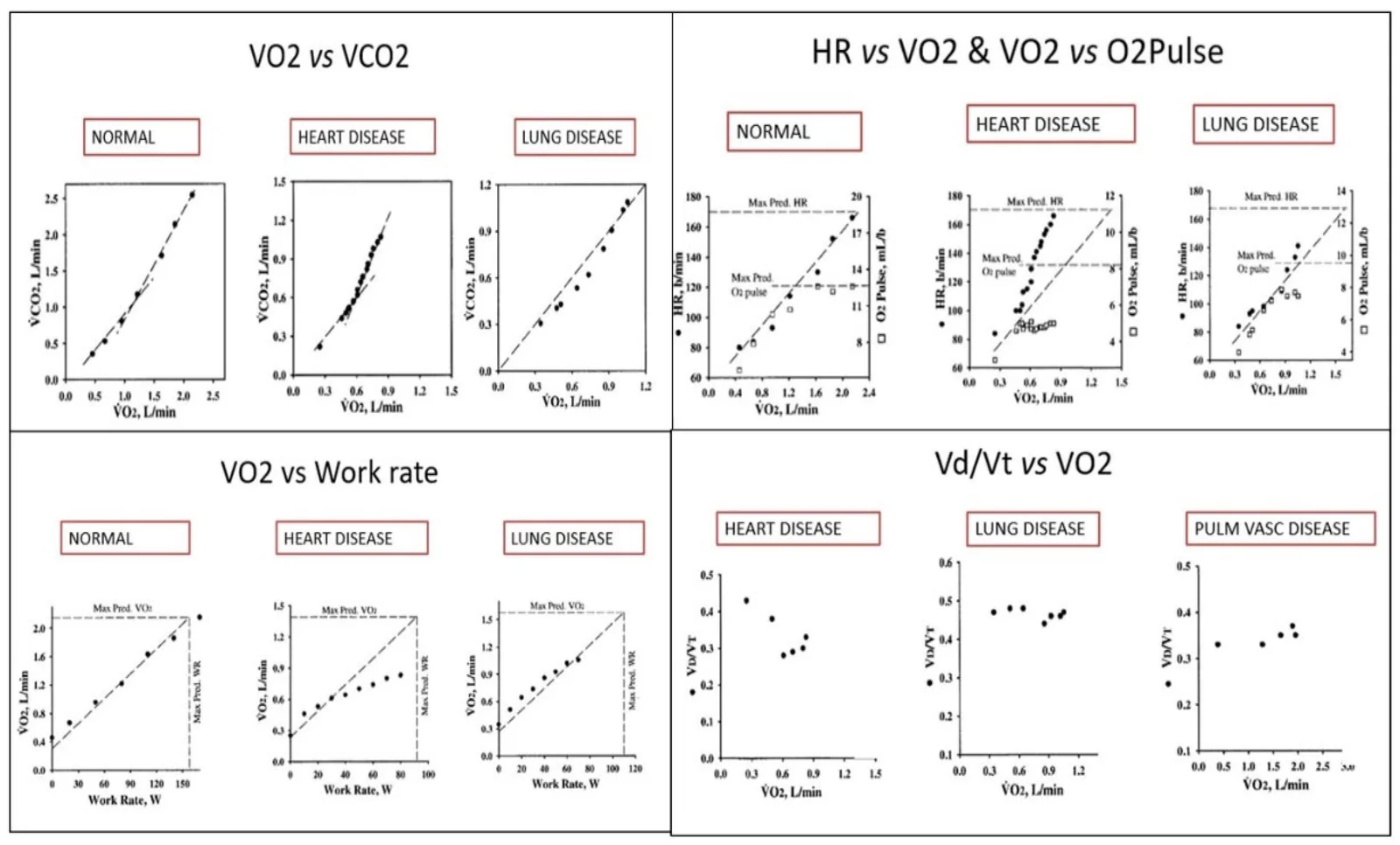
Distinguishing features of cardiac, ventilatory and pulmonary vascular limitation to exercise. Panels show VCO_2_ versus VO_2_; heart rate and O_2_ pulse versus VO_2_; VO_2_ versus work rate; and VD/VT versus VO_2_ in a healthy subject and in patients with heart disease, lung disease and pulmonary vascular disease. Adapted from the ATS/ACCP Statement on Cardiopulmonary Exercise Testing. Am J Respir Crit Care Med. 2003;167(2):211–277. Adapted with permission of the American Thoracic Society. Copyright © 2003 American Thoracic Society.

The features distinguishing ventilatory from cardiac limitation are shown in Figure 8.

## 8. Abnormal CPET Patterns in Other Conditions

Ventilatory, cardiac and gas exchange (pulmonary vascular) limitation to exercise each produce characteristic changes in physiologic variables. Deconditioning and obesity also produce specific patterns on CPET. Recognizing these patterns allows coexisting conditions to be identified in patients with COPD.

### 8.1. CPET Pattern in Cardiac Disease [37,42]

Exercise capacity is reduced, as indicated by a reduced VO_2_peak. Cardiovascular responses are abnormal: HRR is decreased or absent and the O_2_ pulse is reduced, often reaching an early plateau as stroke volume fails to increase. The AT occurs early, at less than 40% of predicted VO_2_max. Ventilatory responses are normal, with a normal BR, a normal maximal RR and a normal PaCO_2_. Gas exchange is often abnormal.

Heart failure is commonly associated with ventilatory inefficiency: VE/VO_2_ and VE/VCO_2_ are increased above the upper limit of normal (for example, a VE/VCO_2_ at the AT above approximately 34; Table 4), and a high VE/VCO_2_ slope carries adverse prognostic significance. This reflects an increased physiological dead space fraction from reduced and heterogeneous pulmonary perfusion, which may be evident at rest and during exercise. Unlike in pulmonary vascular disease, VD/VT usually falls at least partly during exercise (Figure 8).

The oxygen uptake efficiency slope (OUES) is the slope of the relationship between VO_2_ (y-axis) and the logarithm (log_10_) of VE (x-axis) during incremental exercise. It indicates how effectively O_2_ is taken up for a given ventilation and correlates with VO_2_peak. Its main clinical advantage is that it can be calculated from submaximal data and therefore does not depend on the patient exercising to maximal effort, which is useful in COPD, where tests are often terminated early by dyspnea. OUES tends to be reduced in heart failure and to be relatively preserved in COPD [43].

OUES is not a stand-alone diagnostic test for heart failure, but it may contribute to discrimination when interpreted with the other variables. A low OUES, together with a low or early-plateauing O_2_ pulse, a low AT and a preserved BR, in a patient with COPD raises suspicion of additional cardiovascular impairment. This should prompt confirmatory investigation with echocardiography and natriuretic peptide measurement [44]. This matters because unrecognized heart failure was found in about one in five older patients with stable COPD [45]. If heart failure is confirmed, it can then be managed according to its own evidence-based guidelines.

### 8.2. CPET Pattern in Pulmonary Vascular Disease [37,42]

Exercise capacity is reduced, with a reduced VO_2_peak. Cardiovascular responses are abnormal, with a low or absent HRR and a low O_2_ pulse, and the AT may occur early (<40% of predicted VO_2_max). Gas exchange is markedly abnormal: VD/VT is moderately to severely increased at rest and does not fall with exercise; VE/VCO_2_ and VE/VO_2_ at the AT are markedly increased, well above the normal range in Table 4; and there is arterial hypoxemia with a markedly increased P(A-a)O_2_. Ventilatory responses are normal, with a normal or low-normal BR, a maximal RR below 50 breaths/min and a normal PaCO_2_. The VD/VT response in pulmonary vascular disease is shown in Figure 8 [37].

### 8.3. CPET Pattern in Obesity [37,42]

Healthy obese individuals perform more work to move their body mass, but their physiologic responses are often otherwise normal. VO_2_peak in obesity is normal when expressed in L/min but low when expressed in mL/kg/min [43].

### 8.4. CPET Pattern in Deconditioning [37,42]

Exercise limitation due to deconditioning is characterized by a reduced VO_2_peak, which can make it difficult to distinguish from disease-related limitation. HRR may be reduced or absent, the AT may be normal or reduced, and the O_2_ pulse is reduced [17]. There is no evidence of ventilatory or gas exchange abnormality. A low AT is non-specific: it may reflect deconditioning, cardiac disease, skeletal muscle dysfunction or anemia. A low AT without ventilatory limitation suggests potential for a physiological training response, but it does not by itself establish that exercise training is the appropriate treatment. Referral for PR rests on symptoms, functional limitation and established PR indications.

### 8.5. Suboptimal Effort [40,42]

Suboptimal effort may be suspected when exercise stops prematurely with a reduced VO_2_peak and a normal or unattained AT, together with a markedly increased HRR and BR and a low peak RER. Irregular, erratic breathing patterns and RR changes unrelated to the increase in work may suggest a behavioral or volitional cause.

## 9. Utility of CPET in COPD

Dyspnea and exercise limitation in COPD have a major bearing on health-related quality of life, so objective assessment of exercise capacity is valuable in these patients. For most, field tests are sufficient; CPET adds value when the mechanism of exercise limitation is uncertain. Although damage to the small airways and alveoli is the main cause of exercise limitation in COPD, other factors may contribute, including cardiac dysfunction, respiratory and peripheral muscle weakness, pulmonary vascular disease, nutritional imbalance, obesity and deconditioning.

CPET can help determine which of these factors limit exercise, assess the severity of DH and the response to therapy, and estimate the functional reserve of each organ system involved in the exercise response. It is important to distinguish this physiological information from evidence of clinical benefit: to our knowledge, no randomized trials have shown that a CPET-guided management strategy improves patient-centered outcomes in COPD compared with usual care. The applications below should be read in that light.

### 9.1. Clinical Applications of CPET in COPD

1. Assessing functional capacity and exercise intolerance. CPET evaluates the coordinated function of the cardiovascular and respiratory systems during exercise and quantifies exercise limitation by measuring VO_2_. It identifies the severity and mechanisms of dyspnea, which can be disproportionate to resting pulmonary function, particularly in mild COPD [8]. CPET provides an objective evaluation of functional capacity and can help with disability assessment [46].
2. Identifying concomitant causes of dyspnea. CPET can identify other organ dysfunction that contributes to dyspnea and exercise intolerance, distinguishing concomitant cardiovascular and musculoskeletal limitation from the ventilatory limitation of COPD [47]. It helps clinicians understand the specific mechanisms of dyspnea, such as increased ventilatory drive, respiratory muscle dysfunction or impaired gas exchange [9]. Adverse cardiopulmonary interactions, comorbid cardiovascular disease or skeletal muscle dysfunction can impair O_2_ delivery and utilization even at submaximal exercise, producing abnormal VO_2_ responses. CPET can therefore uncover coexisting heart failure, myopathy or deconditioning that may be exacerbating exercise limitation.
3. Prognosis and preoperative evaluation. VO_2_peak and VE/VCO_2_ are associated with mortality in COPD. However, a 2026 systematic review found that the independent prognostic value of VO_2_peak in multivariable models was inconsistent, and evidence for the incremental value of CPET variables over established composite indices remains limited. Routine use of CPET for prognostic stratification is therefore not currently supported, apart from exercise capacity as a component of composite indices such as the body mass index, airflow obstruction, dyspnea and exercise capacity (BODE) index [12]. CPET can be used to assess preoperative risk and the need for perioperative precautions; in surgical candidates with COPD, it can detect myocardial ischemia and measure cardiovascular reserve. For lung resection, earlier guidelines used VO_2_peak thresholds of <10 mL/kg/min (high risk) and >20 mL/kg/min (acceptable risk) [48,49]. The 2025 ERS/ESTS clinical practice guideline recommends CPET particularly for patients with altered pulmonary function and suboptimal results on lower-technology exercise tests. Because of limitations in the available evidence, it does not endorse a single universal CPET cut-off for surgical eligibility. A VO_2_peak below 12 mL/kg/min or a VE/VCO_2_ slope above 40 may suggest increased surgical risk, but these values should be interpreted in the context of the overall multidisciplinary assessment [10]. CPET is also used in the evaluation of candidates for lung volume reduction surgery (LVRS). In chronic heart failure, the prognostic value of VO_2_peak is used to select patients for advanced therapies such as left ventricular assist devices and heart transplantation; a VO_2_peak of ≤12 mL/kg/min in patients tolerating beta-blockers (≤14 mL/kg/min in those intolerant of beta-blockers) supports listing for transplantation [50,51]. CPET can also identify patients who may benefit from enhanced perioperative management, such as supplemental oxygen or preoperative PR.
4. Supporting pulmonary rehabilitation. PR is an effective treatment for COPD, and exercise training is its core component [4,47]. CPET is useful, but not necessary, for PR. PR can be prescribed safely and effectively without CPET. Training intensity can be set from a percentage of the peak work rate on an incremental test, from walking speed derived from field tests such as the 6MWT or ISWT, by targeting a dyspnea or fatigue rating of about 4–6 on the modified Borg scale, or by heart rate in patients without limiting cardiac disease [4,52]. To our knowledge, randomized trials directly comparing CPET-guided prescription with conventionally prescribed PR in COPD are lacking, so superiority of CPET-guided prescription has not been established. In selected patients, CPET provides an objective baseline and helps define exercise intensity. Examples are patients with suspected cardiac comorbidity, exertional arrhythmia or marked desaturation, patients with uncertain safety, patients who respond poorly to an initial program, and patients in whom precise work-rate-based or interval training is planned. Apart from increasing VO_2_peak, exercise training can modify breathing pattern, which may reduce DH and thereby reduce exercise limitation and dyspnea [48].
5. Evaluating treatment efficacy. CPET can be used to monitor the response to treatment, such as bronchodilators or supplemental oxygen, and to evaluate the effectiveness of PR. Constant work rate tests with isotime IC and dyspnea measurements are the most responsive CPET outcomes for this purpose [14]. Changes in CPET variables help clinicians judge the effectiveness of interventions and guide treatment adjustments.
6. Prescribing supplemental oxygen. By documenting exertional desaturation and the work rate at which it occurs, and by allowing repeat testing with supplemental oxygen, CPET can inform decisions about ambulatory oxygen in patients whose field test results are equivocal [30].

### 9.2. Clinical Algorithm

Figure 2 summarizes a practical approach to choosing between field tests and CPET in COPD. Field tests (6MWT or ISWT) are sufficient when the question is routine: quantifying functional capacity, measuring the outcome of PR, screening for exertional desaturation or following disease progression. CPET is indicated when the clinical question requires mechanistic information. Examples are dyspnea or exercise limitation out of proportion to lung function, suspected coexisting cardiac, pulmonary vascular or muscular limitation, preoperative risk stratification, individualized training prescription in complex or high-risk patients, and disability evaluation. CPET is also indicated when field test results do not answer the clinical question or are discordant with symptoms. Contraindications (Table 2) should be reviewed before referral.

### 9.3. Illustrative Case

The following hypothetical case illustrates how CPET can change management. A 68-year-old man, a former smoker, has COPD with moderate airflow limitation (post-bronchodilator FEV_1_/FVC 0.58; FEV_1_ 62% predicted). Despite adherence to dual long-acting bronchodilator therapy, he reports dyspnea on walking 100 m on level ground (modified Medical Research Council grade 3), which appears disproportionate to his spirometry. His 6MWD is 310 m without significant desaturation. The field test confirms reduced functional capacity but does not explain it.

Incremental cycle CPET (10 W/min ramp) is stopped because of dyspnea. VO_2_peak is 11.8 mL/kg/min (52% of predicted). However, BR is preserved (VEmax/MVV 62%) and IC falls by only 0.15 L, indicating that ventilatory mechanics are not the limiting factor. The peak HR is 145 beats/min (HRR 7 beats/min), and the O_2_ pulse plateaus early at 62% of predicted. The AT occurs at 34% of predicted VO_2_max, VE/VCO_2_ at the AT is 39, and OUES is reduced. This pattern indicates cardiovascular rather than ventilatory limitation. Echocardiography shows a left ventricular ejection fraction of 35%, and N-terminal pro-B-type natriuretic peptide is elevated.

Guideline-directed heart failure therapy is started and the patient is enrolled in PR, with the training intensity set from the CPET peak WR. Six months later, a repeat CPET shows a VO_2_peak of 14.5 mL/kg/min and less dyspnea. In this scenario, CPET redirected the diagnostic work-up toward cardiac evaluation. The case illustrates diagnostic reasoning; it does not imply that CPET-guided management improves outcomes, which has not been established in clinical trials.

## 10. Discussion

The aim of this review was to summarize the physiological basis of exercise limitation in COPD and the CPET variables used to characterize it, to describe the CPET patterns that distinguish ventilatory from other causes of exercise intolerance, and to appraise critically the evidence for the clinical applications of CPET in COPD. CPET can contribute to the management of COPD by providing a comprehensive assessment of exercise capacity and by detecting coexisting organ dysfunction.

This review has shown how the mechanical consequences of expiratory flow limitation, chiefly DH and the resulting constraint on VT expansion, together with gas exchange inefficiency, produce a characteristic ventilatory-limitation pattern on CPET. It has also shown how this pattern differs from cardiac, pulmonary vascular, deconditioning and effort-related patterns. Because these mechanisms can coexist in the same patient, and because exertional symptoms often correlate poorly with resting FEV_1_, CPET can explain dyspnea in individual patients in a way that resting tests cannot [8,9]. This mechanistic information can inform management, for example by directing investigation toward a comorbidity such as heart failure, by documenting the response to bronchodilators or oxygen, by helping to set exercise intensity in selected patients, and by contributing to surgical risk assessment. However, it should be distinguished from evidence that CPET-guided strategies improve patient-centered outcomes, which is currently lacking. CPET is best viewed as a selective phenotyping and problem-solving tool rather than a routine investigation.

### 10.1. Comparison with Previous Reviews and Evidence

Our review agrees with previous reviews on several points. CPET is the most complete method for characterizing the multisystem determinants of exercise intolerance in COPD [7,16,20]. Dynamic respiratory mechanics and ventilatory inefficiency are the main physiological correlates of exertional dyspnea [8,9,18], and the test is particularly informative when dyspnea is disproportionate to resting lung function [7,8].

Our interpretation differs from some earlier accounts in three respects. First, in line with recent work, we caution against relying on fixed reference thresholds and single variables, and we favor integrative interpretation against lower limits of normal [15]. Second, the prognostic role of CPET in COPD now appears more limited than earlier narrative reviews suggested. A 2026 systematic review found associations between VO_2_peak, VE/VCO_2_ and mortality, but inconsistent independent and uncertain incremental value over composite indices [12]. Third, some reviews have proposed that a low AT without ventilatory limitation predicts a good response to exercise training [7]. This is physiologically plausible, but in our view a low AT is non-specific and should not by itself determine treatment. Likewise, the preoperative thresholds of earlier guidelines [49] have been replaced by a more nuanced, multidimensional approach [10]. Across these areas, the evidence base consists largely of physiological and observational studies. Trials testing whether CPET-guided management improves patient-centered outcomes are lacking, and this is the main limitation of the current evidence.

### 10.2. Limitations of CPET

CPET has important limitations. The results depend on patient motivation and effort, and many patients with COPD stop before reaching physiological limits. Reference values vary between equations and are less well established for older, obese and non-White populations, so conclusions about “normality” are sensitive to the reference set chosen [13,15]. Interpretation requires expertise; common pitfalls include reliance on a single variable, such as when concluding that ventilation is not limiting because BR is preserved even though mechanical constraints are present, and failing to relate physiological responses to the patient’s symptoms [15]. Non-invasive CPET cannot directly measure cardiac output, pulmonary artery pressure or filling pressures, so invasive CPET may be needed in selected patients. Pulse oximetry may be inaccurate during exercise. Finally, results differ between cycle and treadmill protocols, which complicates comparisons across laboratories [13].

### 10.3. Cost-Effectiveness and Practical Barriers to Implementation

CPET requires dedicated equipment, calibration, trained staff and approximately an hour of laboratory time, and it is more expensive than field walking tests [14]. To our knowledge, no formal cost-effectiveness analyses of CPET in COPD have been published. Its value is greatest where it changes management, for example by identifying a treatable comorbidity, by preventing repeated non-diagnostic investigations in patients with unexplained dyspnea, or by stratifying surgical risk.

For routine monitoring and PR outcome assessment, field tests are adequate and less costly. Barriers to wider use include high costs, limited availability of laboratories, reimbursement that is low relative to the time required, and a shortage of clinicians trained in interpretation [15]. Targeted use guided by an explicit clinical question (Figure 2) is the most efficient way to deploy this resource.

### 10.4. Future Research Directions

Several areas merit further research: (1) contemporary reference values across age, sex, body size and ethnicity, and standardized, individualized WR increments for different lung diseases [13]; (2) interpretive frameworks that integrate dyspnea intensity, operating lung volumes and ventilatory efficiency, which may detect clinically important abnormalities in mild COPD and in symptomatic smokers with preserved spirometry [8,11]; (3) the prognostic value of submaximal and effort-independent indices such as the VE/VCO_2_ nadir and OUES in COPD with cardiovascular comorbidity; (4) simplified or portable systems that could extend CPET beyond specialist laboratories; (5) formal health-economic evaluations of CPET-guided management compared with management based on field tests; and (6) randomized trials testing whether CPET-guided strategies, for example in unexplained dyspnea or PR prescription, improve patient-centered outcomes compared with usual care.

## 11. Conclusions

CPET evaluates the integrated responses of the respiratory, cardiovascular, muscular and metabolic systems during exercise. In COPD it is a powerful tool for selective phenotyping and problem-solving, rather than a routine investigation for every patient. Its value is greatest in unexplained or disproportionate exertional dyspnea, where it can distinguish ventilatory, cardiac, pulmonary vascular and peripheral limitations. It is also valuable in preoperative assessment, interpreted according to current ERS/ESTS guidance, and in selected rehabilitation settings. This positioning is consistent with GOLD 2026, which regards exercise testing as an additional investigation when symptoms and airflow obstruction are discordant [3]. CPET is safe when contraindications are respected, but it is resource-intensive, and it should be interpreted integratively rather than through isolated cut-offs. Whether CPET-guided management strategies improve patient-centered outcomes or resource use remains to be established in prospective trials, which should be a priority for future research.

## Figures and Tables

**Table 1:** Key developments since previous reviews and their clinical implications.

Development	Clinical Implication
ERS standardization of CPET in chronic lung disease (2019) [13]	Standard incremental protocol (8–12 min incremental phase) improves comparability across laboratories and studies
Constant work rate (endurance) testing for interventions (ERS 2016) [14]	More responsive than incremental tests or 6MWT for bronchodilator and rehabilitation effects; isotime IC and dyspnea add mechanistic insight
CPET in mild COPD and out-of-proportion dyspnea (2020) [8]	Identifies gas exchange inefficiency and mechanical constraints despite near-normal FEV_1_
Ventilatory efficiency: slope, nadir and intercept (2021) [11]	In advanced COPD the VE/VCO_2_ slope may fall; nadir and intercept better reflect gas exchange inefficiency
Interpretive pitfalls of CPET (2021) [15]	Integrative interpretation, using lower limits of normal and symptoms, rather than isolated fixed cut-offs
ERS/ESTS guideline on fitness for curative-intent lung cancer treatment (2025) [10]	CPET for patients with altered lung function and suboptimal low-technology test results; VO_2_peak < 12 mL/kg/min or VE/VCO_2_ slope > 40 suggests increased risk; no single universal cut-off
GOLD 2026 report [3]	Exercise testing positioned as an additional investigation when symptoms and airflow obstruction are discordant
Systematic review of prognostic value of CPET in COPD (2026) [12]	VO_2_peak and VE/VCO_2_ associated with mortality, but independent and incremental prognostic value uncertain; routine prognostic use not supported

**Table 2:** Contraindications to CPET.

Absolute	Relative
Acute myocardial infarction (3–5 days)	Left main coronary stenosis or its equivalent
Unstable angina	Moderate stenotic valvular heart disease
Uncontrolled arrhythmia causing symptoms or hemodynamic compromise	Severe untreated arterial hypertension at rest (>200 mmHg systolic, >120 mmHg diastolic)
Syncope	Tachyarrhythmia or bradyarrhythmia
Active endocarditis; acute myocarditis or pericarditis	High-degree atrioventricular block
Symptomatic severe aortic stenosis	Hypertrophic cardiomyopathy
Uncontrolled heart failure	Significant pulmonary hypertension
Acute pulmonary embolus or pulmonary infarction; thrombosis of lower extremities	Advanced or complicated pregnancy
Suspected dissecting aneurysm	Electrolyte abnormalities
Uncontrolled asthma; pulmonary edema; respiratory failure	Orthopedic impairment that compromises exercise performance
Room-air SpO_2_ ≤ 85% at rest (testing may proceed with supplemental O_2_)	
Acute non-cardiopulmonary disorder that may affect, or be aggravated by, exercise (e.g., infection, renal failure, thyrotoxicosis)	
Mental impairment leading to inability to cooperate	

Adapted from the ATS/ACCP Statement on Cardiopulmonary Exercise Testing. Am J Respir Crit Care Med. 2003;167(2):211–277 [37]. In COPD, testing should also be deferred until the patient is clinically stable after an exacerbation.

**Table 3:** Main CPET variables, their physiological meaning and relevance in COPD.

Variable	Physiological Meaning	Relevance in COPD
VO_2_peak	Integrated capacity for O_2_ transport and utilization	Usually reduced; summarizes functional capacity; associated with mortality, but independent prognostic value uncertain [12]
Anaerobic threshold (AT)	Onset of supplementary anaerobic metabolism; integrated index of O_2_ delivery and muscle oxidative capacity	Often normal or indeterminate; a low AT is non-specific (deconditioning, cardiac disease, myopathy, anemia)
Respiratory exchange ratio (RER)	VCO_2_/VO_2_ measured at the mouth	May remain low at peak when ventilatory limitation ends exercise; not a stand-alone effort criterion
Breathing reserve (BR)	Ventilatory capacity remaining at peak exercise (MVV − VEmax)	Low or absent in ventilatory limitation; a preserved BR does not exclude mechanical constraint
Breathing pattern (VT, RR) and IC, if measured	Operating lung volumes and tidal volume expansion	Fall in IC indicates DH; early VT plateau is linked to dyspnea [18]
VE/VCO_2_ (slope, nadir, intercept)	Ventilatory efficiency; determined by VD/VT and PaCO_2_ set point	Increased in mild-to-moderate COPD; slope may fall in advanced disease, so nadir and intercept are also needed [11]
VD/VT	Fraction of each breath that is wasted ventilation	Increased at rest, with a less-than-normal fall during exercise
SpO_2_, PaO_2_, P(A-a)O_2_	Adequacy of pulmonary gas exchange	Exertional desaturation; informs ambulatory oxygen decisions [5]
PETCO_2_	End-tidal CO_2_; reflects dead space and alveolar ventilation	Low with increased dead space; may rise near peak with relative hypoventilation
Heart rate and HRR	Chronotropic response and cardiovascular reserve	HRR often preserved in ventilatory limitation; low HRR with low O_2_ pulse suggests cardiovascular limitation
O_2_ pulse (VO_2_/HR)	Surrogate of stroke volume × arteriovenous O_2_ difference	Usually normal; low or early plateau raises suspicion of cardiac comorbidity
OUES	Effort-independent efficiency of O_2_ uptake relative to ventilation	A low value, with other variables, may raise suspicion of heart failure; not diagnostic on its own
Borg dyspnea and leg discomfort ratings	Symptom intensity in relation to work rate and ventilation	Identifies disproportionate dyspnea and the symptom limiting exercise

Abbreviations as in the main text. Sources: [11,15,20,24,37].

**Table 4:** Commonly cited reference thresholds for physiologic variables measured during CPET.

Variable	Criterion of Normality	Units/Notes
VO_2_max or VO_2_peak	>84% of predicted	% predicted (absolute values in L/min or mL/kg/min)
Anaerobic threshold (AT)	>40% of predicted VO_2_max; wide normal range (40–80%)	% of predicted VO_2_max
Heart rate (HR)	HRmax > 90% of age-predicted	Beats/min
Heart rate reserve (HRR)	A	Beats/min (predicted HRmax − observed HRmax)
Blood pressure (BP) at peak exercise	Systolic < 220 and diastolic < 90	mmHg
O_2_ pulse (VO_2_/HR)	>80% of predicted	% predicted (absolute values in mL/beat)
Breathing reserve (BR; also termed ventilatory reserve)	MVV − VEmax > 11 L/min, or VEmax/MVV × 100 < 85% (wide normal range 72 ± 15%)	L/min or %
Respiratory frequency (RR)	<60	Breaths/min
VE/VCO_2_ at AT	<34	Dimensionless ratio
VD/VT	<0.28; <0.30 for age > 40 years	Dimensionless ratio
PaO_2_	>80	mmHg
P(A-a)O_2_	<35	mmHg

Abbreviations: AT, anaerobic threshold; BR, breathing reserve; HR, heart rate; HRR, heart rate reserve; MVV, maximal voluntary ventilation; O_2_ pulse, oxygen pulse; PaO_2_, arterial partial pressure of oxygen; P(A-a)O_2_, alveolar–arterial oxygen gradient; RR, respiratory rate; VD/VT, dead-space-to-tidal-volume ratio; VE, minute ventilation; VEmax, minute ventilation at peak exercise; VE/VCO_2_, ventilatory equivalent for CO_2_; VO_2_, oxygen uptake. These values are approximate guides only. Normal ranges depend on age, sex, body size, exercise modality, protocol and the prediction equations used; lower limits of normal from appropriate reference equations are preferred, and results should be interpreted integratively in the clinical context rather than against isolated cut-offs [13,15]. Source: ATS/ACCP Statement on Cardiopulmonary Exercise Testing. Am J Respir Crit Care Med. 2003;167(2):211–277 [37]. Reprinted with permission of the American Thoracic Society. Copyright © 2003 American Thoracic Society.

## Data Availability

No new data were created or analyzed in this study. Data sharing is not applicable to this article.

## Abbreviations

6MWT, six-minute walk test; 6MWD, six-minute walk distance; ABG, arterial blood gas; AT, anaerobic threshold; BP, blood pressure; BR, breathing reserve; COPD, chronic obstructive pulmonary disease; CPET, cardiopulmonary exercise testing; DH, dynamic hyperinflation; DLCO, diffusing capacity of the lung for carbon monoxide; EELV, end-expiratory lung volume; FEV_1_, forced expiratory volume in one second; FRC, functional residual capacity; FVC, forced vital capacity; HR, heart rate; HRR, heart rate reserve; IC, inspiratory capacity; ISWT, incremental shuttle walk test; LVRS, lung volume reduction surgery; MVV, maximal voluntary ventilation; O_2_ pulse, oxygen pulse (VO_2_/HR); OUES, oxygen uptake efficiency slope; PaCO_2_, arterial partial pressure of CO_2_; PaO_2_, arterial partial pressure of O_2_; P(A-a)O_2_, alveolar–arterial O_2_ gradient; PEEP, positive end-expiratory pressure; PETCO_2_, end-tidal partial pressure of CO_2_; PETO_2_, end-tidal partial pressure of O_2_; PR, pulmonary rehabilitation; RER, respiratory exchange ratio; RR, respiratory rate; RV, residual volume; SpO_2_, oxygen saturation by pulse oximetry; SWD, shuttle walk distance; TLC, total lung capacity; VCO_2_, carbon dioxide output; VD/VT, dead-space-to-tidal-volume ratio; VE, minute ventilation; VE/VCO_2_, ventilatory equivalent for CO_2_; VE/VO_2_, ventilatory equivalent for O_2_; VO_2_, oxygen uptake; VO_2_max/VO_2_peak, maximal/peak oxygen uptake; V/Q, ventilation–perfusion; VT, tidal volume; WR, work rate.
